# DNA-Methylation Analysis as a Tool for Thymoma Classification

**DOI:** 10.3390/cancers14235876

**Published:** 2022-11-29

**Authors:** Timo Gaiser, Daniela Hirsch, Isabel Porth, Felix Sahm, Philipp Ströbel, Andreas von Deimling, Alexander Marx

**Affiliations:** 1 Institute of Pathology, University Medical Center Mannheim, Medical Faculty Mannheim, Heidelberg University, 68167 Mannheim, Germany; 2 Department of Neuropathology, Institute of Pathology, Heidelberg University Hospital, 69120 Heidelberg, Germany; 3 Clinical Cooperation Unit Neuropathology, German Cancer Consortium (DKTK), German Cancer Research Center (DKFZ), 69120 Heidelberg, Germany; 4 Institute of Pathology, University Medical Center Goettingen, University of Goettingen, 37075 Goettingen, Germany

**Keywords:** thymoma, thymic epithelial tumors, methylation, epigenetics, copy number

## Abstract

**Simple Summary:**

Thymomas are rare malignant epithelial tumors of the thymus. They show a spectrum of microscopic appearances (histotypes) that correlate with the risk of killing patients. The various high- and low-risk thymoma histotypes must be distinguished from each other (i.e., classified) to make correct treatment decisions. However, classification is often difficult, even for expert pathologists, since unequivocal microscopic and genetic features may not be present in each thymoma. Therefore, the current study had the aim to improve the classification of thymomas through the application of a novel method—artificial intelligence-assisted methylation profiling—that measures and compares the absence or presence of methyl groups (a chemical modification of the genetic material, i.e., DNA) across cohorts of tumors. The analysis of 113 thymomas revealed that most cases of each microscopically defined thymoma histotype shared a distinct methylation profile. However, tumor cases with overlapping profiles (‘borderliners’) and cases with quite different profiles (‘outliers’) were also detected. In conclusion, this type of methylation profiling is a valuable new tool to improve therapeutic decision-making through the refined classification of thymomas. It holds promise for identifying new thymoma variants and opening novel, personalized therapeutic perspectives.

**Abstract:**

Background: Thymomas are malignant thymic epithelial tumors that are difficult to diagnose due to their rarity and complex diagnostic criteria. They represent a morphologically heterogeneous class of tumors mainly defined by “organo-typical” architectural features and cellular composition. The diagnosis of thymoma is burdened with a high level of inter-observer variability and the problem that some type-specific morphological alterations are more on the continuum than clear-cut. Methylation pattern-based classification may help to increase diagnostic precision, particularly in borderline cases. Methods and Results: We applied array-based DNA methylation analysis to a set of 113 thymomas with stringent histological annotation. Unsupervised clustering and t-SNE analysis of DNA methylation data clearly segregated thymoma samples mainly according to the current WHO classification into A, AB, B1, B2, B2/B3, B3, and micronodular thymoma with lymphoid stroma. However, methylation analyses separated the histological subgroups AB and B2 into two methylation classes: mono-/bi-phasic AB-thymomas and conventional/“B1-like” B2-thymomas. Copy number variation analysis demonstrated methylation class-specific patterns of chromosomal alterations. Interpretation: Our study demonstrates that the current WHO classification is generally well reflected at the methylation level but suggests that B2- and AB-thymomas are (epi)genetically heterogeneous. Methylation-based classifications could help to refine diagnostic criteria for thymoma classification, improve reproducibility, and may affect treatment decisions.

## 1. Introduction

Thymoma is the most common mediastinal tumor in adults but counts among the rarest human tumors, making pathological classification difficult, especially for low-volume centers [1,2]. In addition, the great morphological complexity and heterogeneity of some thymoma subtypes pose diagnostic problems and led to proposals aiming at replacing the World Health Organization (WHO) classification system and terminology [3,4] with a more simplified classification and nomenclature [5,6,7]. However, the striking results of The Cancer Genome Atlas (TCGA) effort based on thymoma cases classified by a panel of expert pathologists [8] strongly reinforced the WHO thymoma classification, which is in worldwide use [9]. The WHO classification distinguishes six common thymoma subtypes: A, including atypical A-thymoma as a variant, AB, B1, B2, B3, and micronodular thymoma with lymphoid stroma (MNT), as well as the exceedingly rare metaplastic thymoma that was neither included in the TCGA study nor the current analysis. Thymomas are classified based on variably pronounced “organo-typical” architectural features (e.g., the presence or absence of ‘medullary islands’ and ‘perivascular spaces’), tumor cell morphology (spindly versus polygonal), and the relative abundance of non-neoplastic immature T cells compared to neoplastic epithelial cells. Given this morphological complexity, it is not surprising that inter-observer reproducibility has remained an issue in real-life settings [10,11,12] and made some authors argue in favor of a simplified histological scheme [7]. On the other hand, meticulous immunohistochemical analysis of WHO-defined thymoma subtypes in terms of functional molecules of the cortical and medullary thymic epithelial cells [13] demonstrated the value of the historic classification by Marino and Müller-Hermelink [14], separating thymomas on the basis of the presumed “cell of origin” into two main categories, cortical and medullary thymomas. Since DNA methylation patterns reflect both the cell type of origin as well as acquired changes during differentiation and tumor development [15], we presumed that DNA methylation patterns could be a new tool for ‘objective’ thymoma subtyping. Surprisingly, many thymoma methylation projects, including TCGA methylation profiling, failed to correlate methylation data with the established WHO histologic subtypes [8]. While TCGA methylation profiling could distinguish thymic squamous cell carcinomas from thymomas [16], it showed no association with the above-mentioned histological WHO classification of thymomas [8]. Since then, however, artificial intelligence has revolutionized the analysis of methylation profiles: the use of deep-learning algorithms has allowed methylome-based cancer diagnosis at such high sensitivity and specificity that methylation profiling has become a commonplace and often indispensable diagnostic tool in the field of brain tumor classification [17], strongly endorsed by the WHO [18]. Furthermore, methylation profiling holds great promise to support the differential diagnosis of cancers with overlapping morphological features, as recently demonstrated for sarcomas [19]. In the present work, we subjected clearly annotated thymoma subtypes to this advanced methylation profile analysis and investigated whether the current WHO-based thymoma subclassification can be retraced by DNA methylation profiling. We also aim to provide a broadly available diagnostic platform to increase diagnostic reproducibility in thymoma pathology.

## 2. Material and Methods

### 2.1. Sample Selection and Quality Control

Formalin-fixed paraffin-embedded (FFPE) tissue was obtained from the Institute of Pathology, University Medical Center Mannheim, Germany. The Mannheim Institute of Pathology, with its director (Prof. Dr. Alexander Marx) and vice director (Prof. Dr. Timo Gaiser), serves as a reference laboratory for mediastinal pathology and has, therefore, outstanding experience in thymoma classification. The thymoma classification was performed in accordance with the current WHO classification [3]. The study was approved by the local ethics committee of the Medical Faculty Mannheim of the University of Heidelberg, Germany (2012-608R-MA).

Sections of the FFPE samples selected for methylation array analysis were mounted on glass slides (by default, 10 μm thick, 8 consecutive slides). On a consecutive H&E-stained section (3–4 μm), a suitable tumor area was identified by TG or AM. A tumor content of at least 80% was selected where possible, and non-neoplastic tissue, blood, or excessive areas of necrosis were excluded.

### 2.2. DNA Extraction and Quantification

Slides with mounted tissue were dewaxed (3 washes in xylene and 2 washes with industrial methylated spirit) and air-dried. Tissue selected for the analysis was scraped off and collected in lysis buffer, and DNA was extracted with the Maxwell 16 Lev FFPE DNA Purification Kit on a Maxwell 16 extractor. The DNA extraction procedure was carried out according to manual #TM349 for DNA extraction (Promega, Madison, WI, USA). DNA was then quantified, and A260/A280 ratios were determined on a Nanodrop 8000 spectrophotometer (ThermoFisher, Waltham, MA, USA). An A260/A280 ratio of ~1.8 was considered to represent sufficient purity to proceed with the methylation study.

### 2.3. Methylation Array Processing and Copy Number Profiling

All thymoma samples were submitted for DNA methylation analysis. The Illumina Infinium Human Methylation EPIC (850K) BeadChip array (Illumina, San Diego, CA, USA) was employed, following the manufacturer’s instructions. Copy number profile (CNP) analysis was assessed by R package “conumee” [20] after additional baseline correction (https://github.com/dstichel/conumee, last accessed on 28 November 2022).

### 2.4. Statistical Analysis

DNA methylation data were processed with the R/Bioconductor package “minfi” (version 1.20) [21]. The t-SNE plot was computed by the R package “Rtsne” from 20,000 most variable CPG sites across the dataset, 3000 iterations, and a perplexity value of 20.

## 3. Results

### 3.1. Thymoma Methylation Classes and Histological Characteristics

We examined the methylation profiles of nine normal thymus samples and 113 prototypical thymomas belonging to the six main WHO-defined histological subtypes: WHO type A-thymoma (A, n = 9), atypical type A-thymoma (AA, n = 5), AB-thymoma (AB, n = 24), B1-thymoma (B1, n = 9), B2-thymoma (B2, n = 33), B3-thymoma (B3, n = 7), and micronodular thymoma with lymphoid stroma (MNT, n = 21). We also included the most common composite thymoma containing more than one subtype, i.e., type B2/B3-thymoma (B2/B3, n = 5). For DNA methylation profiling, the Illumina MethylationEPIC (850K) array platform was used as described previously [19]. Following quality control, methylation data were analyzed by t-Distributed Stochastic Neighbor Embedding (t-SNE), identifying groups of tumors sharing methylation patterns (methylation classes).

**Figure 1:**
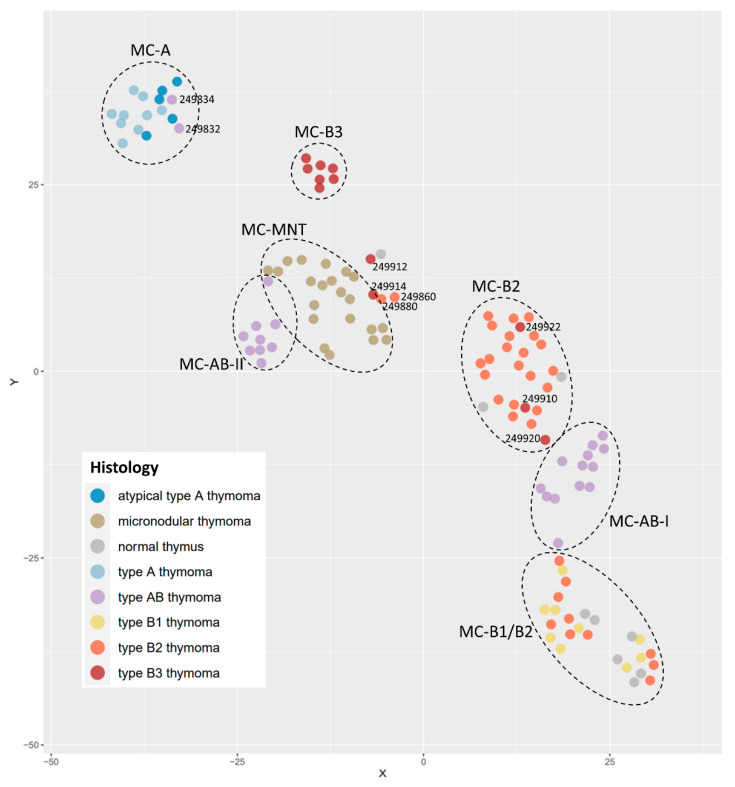
DNA methylation-based thymoma classification colored by the histological diagnosis. Circles indicate seven different methylation groups (MC-A; MC-AB-I; MC-AB-II; MC-B1/B2; MC-B2; MC-B3; and MC-MNT) established by t-Distributed Stochastic Neighbor Embedding (t-SNE) dimensionality reduction. Each of the MC-groups is mainly composed of one histological subgroup (displayed by different colors) despite some remarkable features: 1. A-thymomas cluster together with atypical A-thymomas; 2. AB-thymomas are separated into two clusters that were characterized histologically by a rare monophasic and prototypic bi-phasic growth pattern, respectively; and 3. B2-thymomas are separated into two clusters that were characterized histologically by prototypic B2 and “B1-like” B2-thymomas, respectively. MC-MNT forms a separate methylation entity but has poorly defined boundaries. Normal thymus samples clustered mainly with B1/B2 thymomas, which most closely resemble normal thymus cytologically and architecturally.

Methylation classes (MC) and histologically defined subtypes exhibited a striking overlap. The above-mentioned histological thymoma samples formed seven distinct MC groups: MC-A; MC-AB1; MC-AB2; MC-B1/B2; MC-B2; MC-B3; and MC-MNT. Only the methylation cluster of micronodular thymomas (MC-MNT) was blurred but still formed a distinct group (Figure 1). Normal thymus samples clustered mainly with B1/B2 thymomas, which most closely resemble normal thymus cytologically and architecturally. The following conclusions could be drawn from these analyses: firstly, the initial WHO diagnosis and the methylation groups matched perfectly in 104 out of 113 cases; secondly, five composite thymomas consisting of B2 and B3 components (249910, 249912, 249914, 249920, and 249922) clustered accordingly between MC-B2 and MC-B3; and finally, AB- and B2-thymomas formed two different MC clusters each, designated MC-AB-I (13 cases) and MC-AB-II (9 cases), and MC-B2 (21 cases) and MC-B1/B2 (10 cases). As indicated by its name, the “MC-B1/B2” cluster was formed by B1-thymomas together with a subgroup of B2-thymomas (“B1-like” B2-thymomas) (Figure 1).

**Figure 2:**
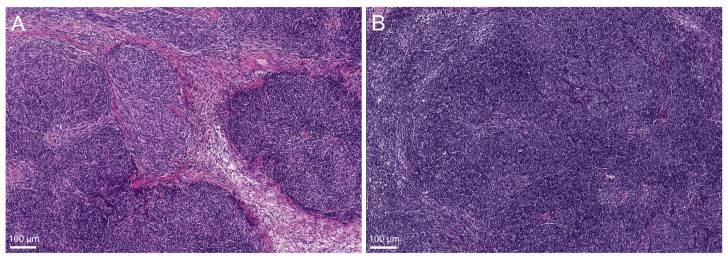
(**A**) Biphasic AB-thymoma with pure spindle cell-rich tumor areas (center) next to lymphocyte-rich type B-like areas (periphery) (hematoxylin and eosin staining). (**B**) Monophasic AB-thymoma with a high lymphocyte content among spindle tumor cells throughout the tumor (hematoxylin and eosin staining).

A retrospective histological review based on these methylation findings revealed different morphologic features in cases from these subgroups. MC-AB-I and MC-AB-II contained AB-thymomas with different lymphocyte contents. Thymomas in the MC-AB-I cluster showed a ‘classical’ biphasic growth pattern, with separated spindle cell-rich type A areas and lymphocyte-rich type B-like areas (Figure 2A). In contrast, the AB-thymoma of the MC-AB-II group exhibited a monophasic morphology with a high lymphocyte content throughout the tumor (Figure 2B).

**Figure 3:**
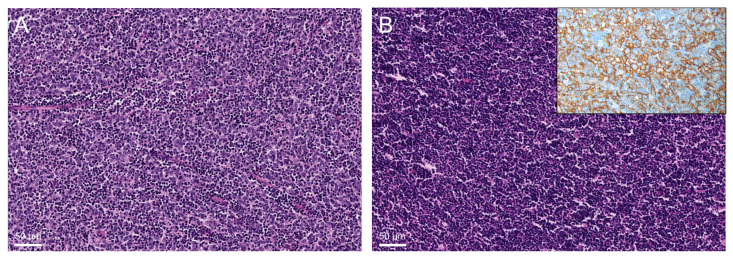
(**A**) “Conventional B2-thymoma” with clusters of neoplastic epithelial cells with vesicular nuclei and distinct nucleoli among numerous lymphocytes (hematoxylin and eosin staining). (**B**) “B1-like” B2-thymoma: While CK19 immunohistochemistry shows a dense epithelial cell network (inlay) not compatible with B1-thymoma, the tumor consists predominantly of lymphocytes with inconspicuous epithelial cells; also note the absence of ‘medullary islands’ which would be required for the diagnosis of B1-thymoma (hematoxylin and eosin staining).

B2-thymomas were also, by methylation, divided into two distinct subgroups: MC-B2 (21 cases) and MC-B1/B2 (10 cases). Histological assessment revealed that MC-B2 thymomas showed high numbers of epithelial cells and easily visible tumor cell clusters (Figure 3A), while MC-B1/B2 thymomas were dominated by thymocytes (Figure 3B). Nevertheless, the diagnostic criteria for B1 thymomas (resemblance to a normal thymus, obvious medullary islands, and a lack of epithelial cells) were not met.

Surprisingly, atypical and conventional A-thymomas formed a single distinct methylation class (MC-A) (Figure 1). Since atypical A-thymomas resemble B3-thymomas in many ways [22], one might have expected clustering closer to B3-thymomas, which was obviously not the case.

Lastly, it is remarkable that micronodular thymomas with lymphoid stroma formed a distinct cluster (MC-MNT) that was clearly separated from the clusters comprising A-thymomas and AB-thymomas, although these three thymoma subtypes share morphologic, immunohistochemical, and genetic features [8]. The different methylation profiles could therefore indicate an important contribution of the non-neoplastic lymphoid stroma to the individual “bulk methylation profile”. In particular, we speculate that the near-absence of a lymphoid stroma in A-thymomas, the variable abundance of immature T cells in AB-thymomas, and the dominance of numerous mature B cells and T cells in the stroma of MNTs may have caused different footprints in the methylation profiles of these tumor entities.

Of note, no association with gender was observed (Supplementary Figure S1A), but interestingly, the distribution of older (>61.5 years) and younger (<61.5 years) individuals was skewed between the MC groups. While in accordance with the literature, younger patients were mainly affected by type B1/B2 thymomas, older patients more frequently developed type A thymomas (Supplementary Figure S1B).

Reassuringly, heatmap analysis recapitulated the methylation differences in the distinct thymoma histotypes identified by t-SNE analysis (Supplementary Figure S2).

### 3.2. Case-by-Case Discussion for “No Match” Samples

**Figure 4:**
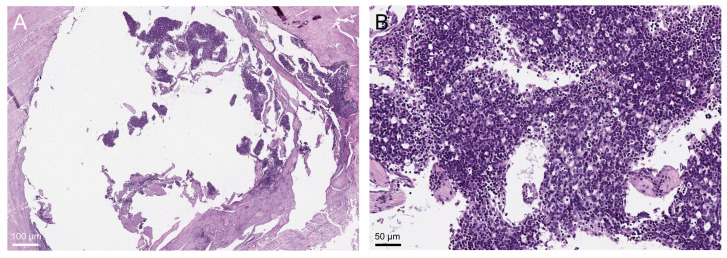
Pseudocystic B2-thymoma showing impressive regressive changes, sclerosing, and inflammatory infiltration (**A**) but also a circumscribed clear B2-thymoma area (**B**).

While methylation profiling assigned almost all thymoma samples to distinct MCs, eight cases (9/113; 8%) were ‘outliers’, i.e., they did not cluster within a distinct MC group or did not cluster in the histologically anticipated MC group. For the following five cases not falling into a distinct or their anticipated MC group, clarifying the discrepancy was easy: all of them (249910, 249912, 249914, 249920, and 249922) were heterogeneous thymomas with B2 and B3 components, making their intermediate position between MC-B2 and MC-B3 plausible. Two B2-thymomas (249860 and 249880) clustered near the MC-MNT group. Microscopically, both tumors showed a circumscribed clear B2-thymoma area that was altered by (pseudocystic) regression, sclerosing, and inflammatory changes, possibly modifying the methylation pattern (Figure 4).

**Figure 5:**
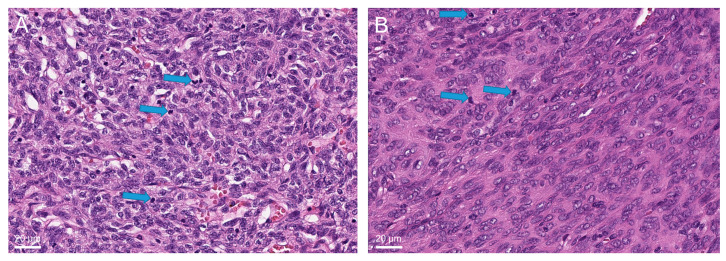
Lymphocyte-poor (type A-like) AB-thymoma (hematoxylin and eosin staining); sparse intratumorous lymphocytes are marked exemplarily with arrows ((**A**), case 249832, (**B**), case 249834). In such cases, the distinction from type A-thymoma is difficult and arbitrary.

Two other cases did not cluster in the histologically anticipated MC group. Both cases (249832 and 249834) were AB-thymomas that clustered with the MC-A cohort. Retrospective histological re-analysis of these “type A-like” cases showed a very low lymphocyte content, making A-thymoma a reasonable differential diagnosis (Figure 5).

### 3.3. Copy Number Profiling of Thymomas

**Figure 6:**
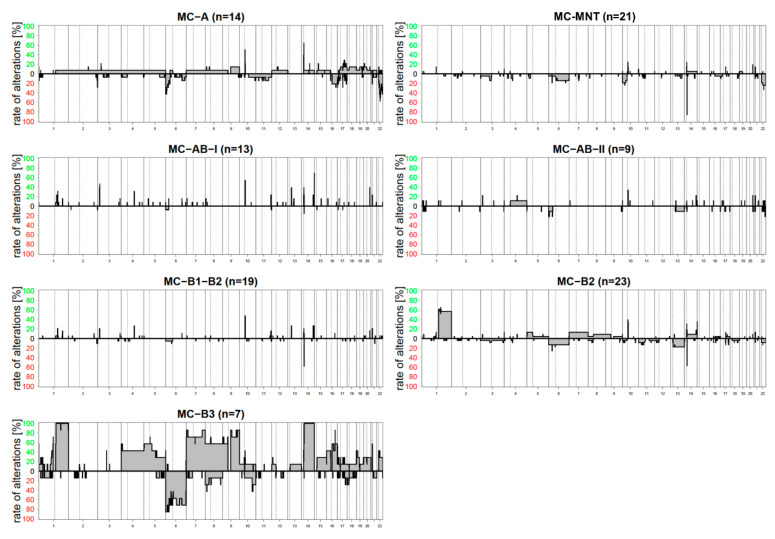
Frequency plots of genomic copy number aberrations (CNAs) in the methylation cluster (MC)-based thymoma subgroups. CNAs are rare in MC-A, MC-MNT, MC-AB-I, MC-AB-II, and MC-B1-B2, but enriched in MC-B2 and MC-B3 thymomas. If present, most CNAs are large-scale, chromosome arm level CNAs. Gains of chromosomal material are most common for chromosome arm 1q in MC-B2, accompanied by 4, 5, 7, 8, 9, 14, and 16 in MC-B3. Deletions most frequently involve chromosome 6.

We generated copy number variation (CNV) plots from all thymomas based on MC- and histological subgrouping (Figure 6 and Figure S3). Summary copy number plots (SCNP) were generated by overlaying individual CNV plots from the distinct subgroups. While histological vs. MC-based SCNPs were concordant, different MC/histology groups exhibited remarkable differences (Figure 6). A-thymoma and atypical A-thymoma exhibited few numerical alterations with no consistently recurrent changes. The impression that atypical A-thymomas exhibit more alterations is deceptive because it was caused by a single case with higher chromosomal instability. MC-MNT, including all cases of micronodular thymoma, showed no recurrent alterations. Similar findings were observed for MC-AB-I and MC-AB-II. All tumors in MC-B1/B2, including all B1 and a fraction of B2-thymomas (“B1-like” B2-thymomas), exhibited a low number of alterations, too. In contrast, MC-B2 (conventional B2-thymoma) showed more alterations with a gain of chromosomal arm 1q in more than half of the cases. MC-B3 was characterized by the highest number of copy number alterations, with gains of chromosomes 1q and 14q in virtually all cases, followed by gains of chromosomes 7, 9, 8, 16, 5, and 4. Losses most frequently involve chromosome 6.

## 4. Discussion

We here demonstrate the successful classification of thymomas by methylation patterns based on array-generated DNA methylation data. Specifically, we show that the current WHO subtyping of thymomas is substantially reflected by our methylation classifier. Furthermore, some unexpected relationships between histological subtypes became obvious: (a) A-thymomas cluster together with atypical A-thymomas; (b) AB-thymomas were separated into two groups, namely the classical biphasic and the less common monophasic AB variant; (c) B2-thymomas were also separated into two subgroups, one of which is epithelial-rich (conventional) and the other constituting “B1-like” lymphocyte-rich B2-thymomas; (d) thymomas consisting of B2 and B3 components clustered between MC-B2 and MC-B3; and (e) A-/AB-thymomas and micronodular thymomas with lymphoid stroma formed distinct methylation groups.

### 4.1. (a) A-Thymomas Cluster Together with Atypical A-Thymomas

The 2015 version of the WHO classification system introduced atypical A-thymoma as a variant of A-thymoma [3] to account for a small subgroup of A-thymomas that exhibit hypercellularity, increased mitotic counts, and foci of necrosis [22]. In the current WHO classification, atypical A-thymoma was established as an A-thymoma subtype [9]. However, only necrosis appears to show prognostic significance [23,24]. On the other hand, distant metastasis in A-thymomas was associated with recurrent genetic aberrations independent of atypical histological features [25], making it debatable whether a distinction between conventional and atypical A-thymomas is clinically warranted. Our data here underline the close relationship of both *morphologically* defined A-thymoma subtypes. Future comparisons of *clinically* aggressive (advanced, unresectable, and lethal) and indolent A-thymomas should therefore increasingly focus on genomic and methylomic differences to identify more meaningful biomarkers instead of morphological features.

### 4.2. (b) Methylation Analyses Separate AB-Thymomas into Two Subgroups: Monophasic and Bi-Phasic AB-Thymoma

One of the most interesting findings presented here was the separation of AB-thymoma into two distinct subgroups. The growth pattern of most AB-thymomas is biphasic, with separated spindle cell-rich type A and lymphocyte-rich type B-like areas in highly variable proportions [9]. However, there are also monophasic AB-thymomas that lack a lymphocyte-poor type A component but are characterized by high numbers of lymphocytes among epithelial spindle cells throughout the tumor [26,27]. So far, there are no established clinical differences between these two subgroups of AB-thymoma, and genetic data is extremely sparse [26,27]. However, the results presented here suggest that it might be relevant to further characterize these two subgroups clinically and at the molecular level.

### 4.3. (c) Methylation Analyses Separate B2-Thymomas into Two Subgroups: Conventional and “B1-like” B2-Thymomas

The two methylation clusters were associated with different thymocyte/epithelial cell ratios on histology. It is well known that the proportion of epithelial cells can vary widely between B-thymomas [9]. Interestingly, one B2-thymoma subgroup (termed “B1-like” B2-thymomas) clustered together with the B1-thymomas, while the other B2-thymoma cases formed a distinct cluster distant from MC-B1/B2. It is noteworthy that both B2-thymoma subgroups did not show medullary islands, therefore ruling out true B1-thymomas. Nevertheless, the current data suggest that future studies should clarify whether B2-thymomas from the MC-B1/B2 cluster have the same indolent clinical course as the vast majority of B1-thymomas [9]. If confirmed, the criteria for the diagnosis of B1-thymomas and B2-thymomas may need reconsideration and refinement. Such an adjustment of diagnostic criteria would be clinically relevant because non-encapsulated B2-thymomas classified according to current WHO criteria are common candidates for adjuvant radiotherapy even after radical resection, while radiotherapy is generally not recommended for completely removed locally invasive B1-thymomas [28].

### 4.4. (d) Composite Thymomas Consisting of B2 and B3 Components Clustered Accordingly between MC-B2 and MC-B3

Five thymomas were initially diagnosed as ‘composite thymomas’ consisting of a mixture of distinct B2 and B3 areas. This histological heterogeneity is a very common finding [7], and it is interesting that methylation analyses were able to recapitulate this heterogeneity by plotting these samples between MC-B2 and MC-B3.

This apparently high sensitivity and discriminatory power of methylation profiling could be meaningful in two settings. Firstly, it could help to improve the clinically meaningful recognition of heterogeneous B2/B3-thymomas that were previously shown to have a relatively favorable or poor prognosis depending on whether the proportion of the B3-component was below or above 10% [7,29]. Secondly, the high discriminatory power can potentially discover new biological tumor entities that are currently counted among the major thymoma histotypes [30]. In the field of non-thymic cancers, the discovery of new tumor entities through methylation profiling has already opened up new therapeutic perspectives, as demonstrated for histologically identical-looking brain tumors [31] and sarcomas [19].

### 4.5. (e) A-Thymoma and Micronodular Thymoma with Lymphoid Stroma form Distinct Methylation Groups Distant from Each Other

MNT has been considered to be closely related to type A-thymoma due to histologic and immunohistochemical similarities between the spindle epithelial cells found in both entities [32]. In addition, extensive areas resembling A-thymoma are found in up to 30% of heterogeneous MNT [33]. Finally, the highly characteristic L424H mutation of the *GTF2I* gene is encountered in both MNT and A-thymomas [8]. Despite these striking similarities, our results suggest that the hyperplastic B cell-rich lymphoid stroma present in all MNTs—a major criterion for the diagnosis—may have shifted the overall methylation pattern to a cluster that is clearly separated from the methylation cluster of A-thymomas.

Calculation of CNV was possible in all thymomas studied here. In line with TCGA findings [8] and historic CGH studies [34,35,36], our analysis revealed an overall low prevalence of CNVs in thymomas, with the highest CNV load in B3-thymomas followed by B2-thymomas (the conventional subtype). Of note, the B2-thymoma subgroup within the MC-B1/B2 cluster (B1-like B2-thymomas) showed barely any CNVs, as did ‘true’ B1 thymomas. This could be another hint that the MC-B1/B2-thymomas could pursue a more indolent clinical course. The CNVs in the different thymoma subgroups consisted mainly of gains and losses of whole chromosomes or chromosome arms. The gain of 1q represented the most frequent genomic alteration across all thymoma subtypes, followed by gains of chromosomes 14, 7, 9q, 5p, and 4, and losses of chromosome 6. Significantly, atypical A-thymomas did not show more alterations than conventional A-thymomas, except for one aberrant case with multiple CNVs.

Our study has limitations. Clustering based on methylation profiles may simply reflect different ratios of thymocytes to epithelial cells. In fact, our findings strongly suggest an impact of the non-neoplastic stroma on the overall methylation profile of a given thymoma. However, determining these ratios is one of the diagnostic cornerstones of the current WHO classification. Moreover, we observed strong clustering differences between thymoma subtypes with similar ratios of thymocytes to epithelial cells (such as thymocyte-poor A-thymomas vs. B3-thymomas or thymocyte-rich AB-thymomas vs. B2-thymomas). The latter cases suggest a high sensitivity of our approach in identifying specific tumor cell methylation profiles even against a background of abundant non-neoplastic thymocytes.

In summary, our study is the most comprehensive methylation analysis of thymomas to date, generally supporting the current WHO classification. Methylation analysis points towards refinements regarding a fraction of B2-thymomas sharing molecular features with B1-thymomas, with potential for clinical consequences.

## Data Availability

Data have been uploaded to the GEO database, and access will be provided upon acceptance of the manuscript.

## Supplementary Materials

The following supporting information can be downloaded at: https://www.mdpi.com/article/10.3390/cancers14235876/s1, Figure S1: DNA methylation-based thymoma classification established by t-Distributed Stochastic Neighbor Embedding (t-SNE) dimensionality reduction, Figure S2: Methylation-based clustering of the different thymoma types shows a decent separation according to the different thymoma histotypes whereby particularly type B1- and B2-thymomas (especially B1-like type B2-thymomas) intermingle, Figure S3: Frequency plots of copy number alterations in thymomas grouped according to histologic subtype.
