# Supplementary material for: DNA-Methylation Analysis as a Tool for Thymoma Classification
Source: Cancers (Basel). 2022 Nov 29;14(23):5876. doi: 10.3390/cancers14235876 (PMC9738683; doi:10.3390/cancers14235876)
Supplement: Supplementary file 1 [file cancers-14-05876-s001.zip › cancers-1971900-supplementary.pdf]

Supplementary Figures

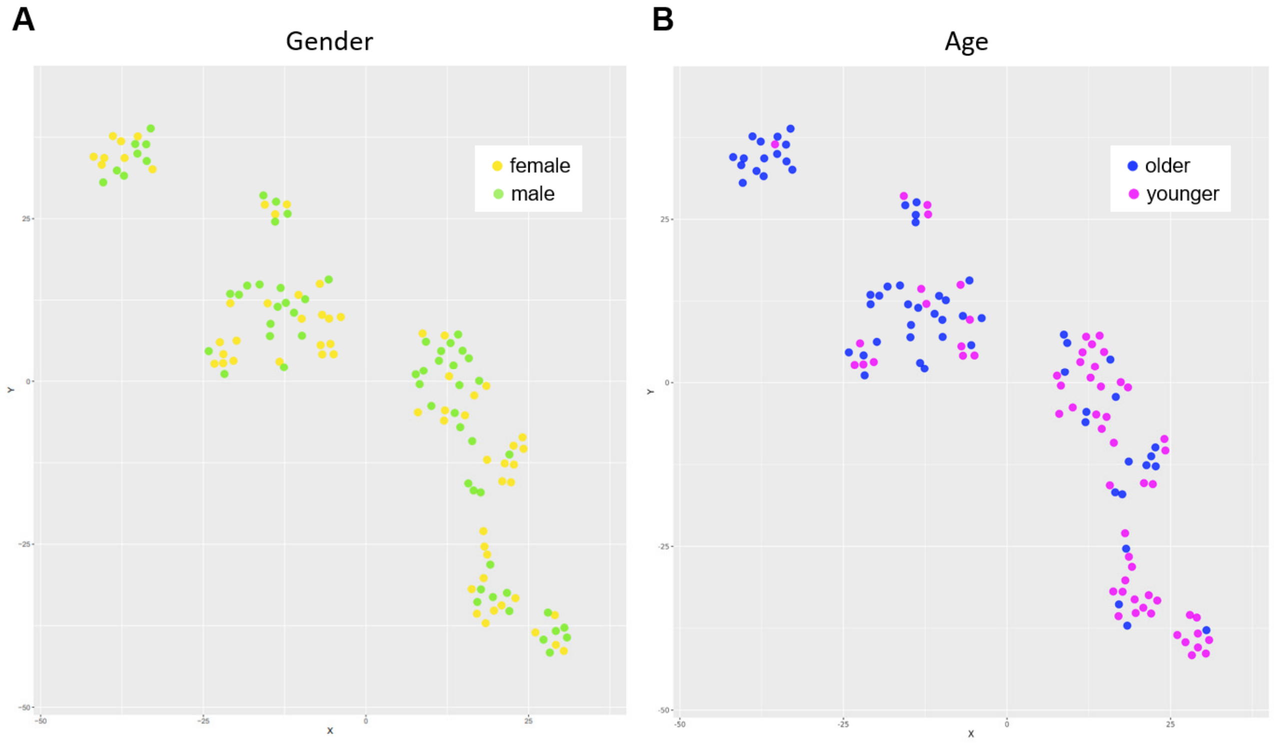

Figure S1. DNA methylation-based thymoma classification established by t-distributed stochastic neighbor embedding (t-SNE) dimensionality reduction. Colored by gender (A) and age (B). (A) DNA methylation-based thymoma classification does not show any gender bias. (B) DNA methylation-based thymoma classification showed that younger (<61.5 years) and older (>61.5 years) patients are not equally distributed among the different methylation clusters. Cluster MC-A harbors mainly older patients while MC-B1/B2 and MC-B2 contain mainly younger patients. This finding is in line with the literature that B1/B2 thymomas occur more frequently in younger patients while older patients more often develop A thymomas.

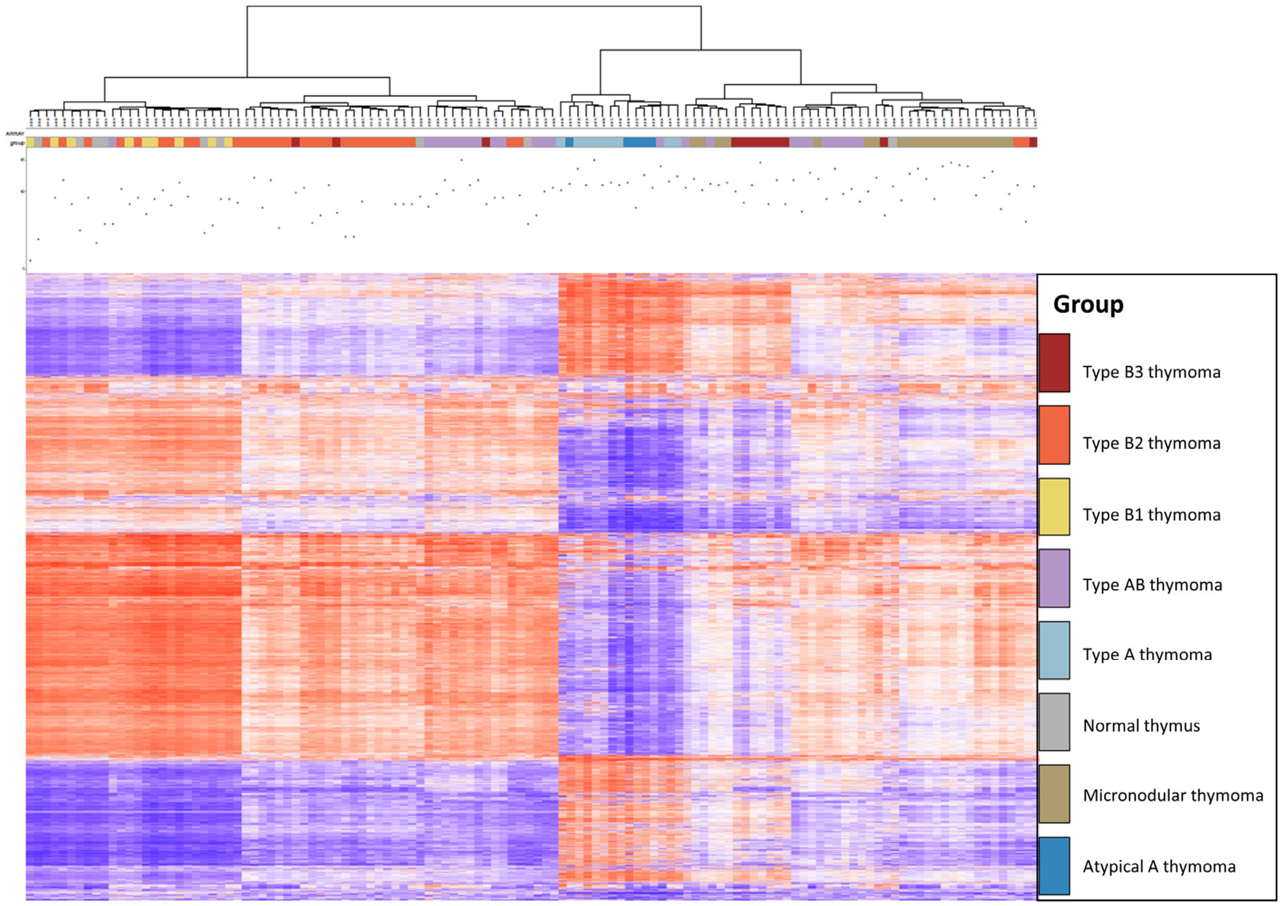

Figure S2. Methylation-based clustering of the different thymoma types shows a decent separation according to the different thymoma histotypes whereby particularly type B1 and B2 thymomas (especially B1-like type B2 thymomas) intermingle.

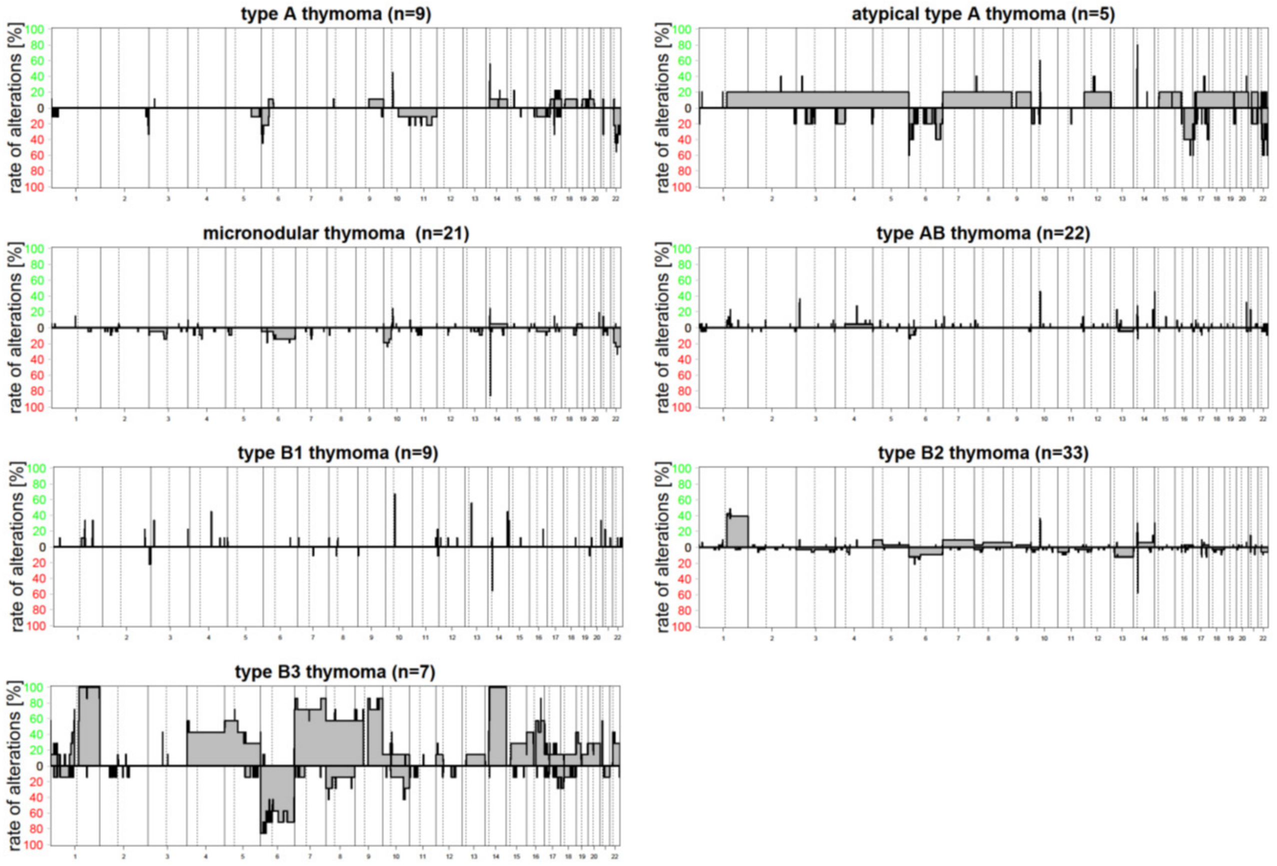

Figure S3. Frequency plots of copy number alterations in thymomas grouped according to histologic subtype.
